# Supplementary material for: Phenome-Wide Analysis of Coffee Intake on Health over 20 Years of Follow-Up Among Adults in Hong Kong Osteoporosis Study
Source: Nutrients. 2024 Oct 18;16(20):3536. doi: 10.3390/nu16203536 (PMC11509949; doi:10.3390/nu16203536)
Supplement: Supplementary file 1 [file nutrients-16-03536-s001.zip › nutrients-3251869-supplementary.pdf]

Supplementary Material

Phenome-Wide Analysis of Coffee Intake on Health over 20 Years of Follow-Up Among Adults in the Hong Kong Osteoporosis Study

Table of Contents

Supplementary Figure S1. Flowchart of sample selection. 2

Supplementary Figure S2. Histogram of the number of cups of coffee intake in the Hong Kong Osteoporosis Study. 3

Supplementary Figure S3. Subgroup analysis for the association between any coffee intake and the 10 top identified phecode diagnoses at FDR <0.05. 4

Supplementary Figure S4. Survival probability stratified by daily coffee consumption 5

Supplementary Figure S5. Manhattan plot for the phenome-wide analysis of coffee intake categories on disease outcomes. 6

Supplementary Table S1. ICD-9 codes used to define the diseases selected from the literature. 7

Supplementary Table S2. Sample characteristics by coffee intake categories. 8

Supplementary Table S3. Subgroup analysis for the association between any coffee intake and the top identified phecode diagnoses. 9

Supplementary Table S4. Association between any coffee intake and specific diseases selected from the literature. 13

Supplementary Table S5. Association between any coffee intake and the top identified phecode diagnoses after excluding the first 2 years of follow-up. 14

Supplementary Table S6. Association between any coffee intake and all-cause mortality after excluding the first 2 years of follow-up 15

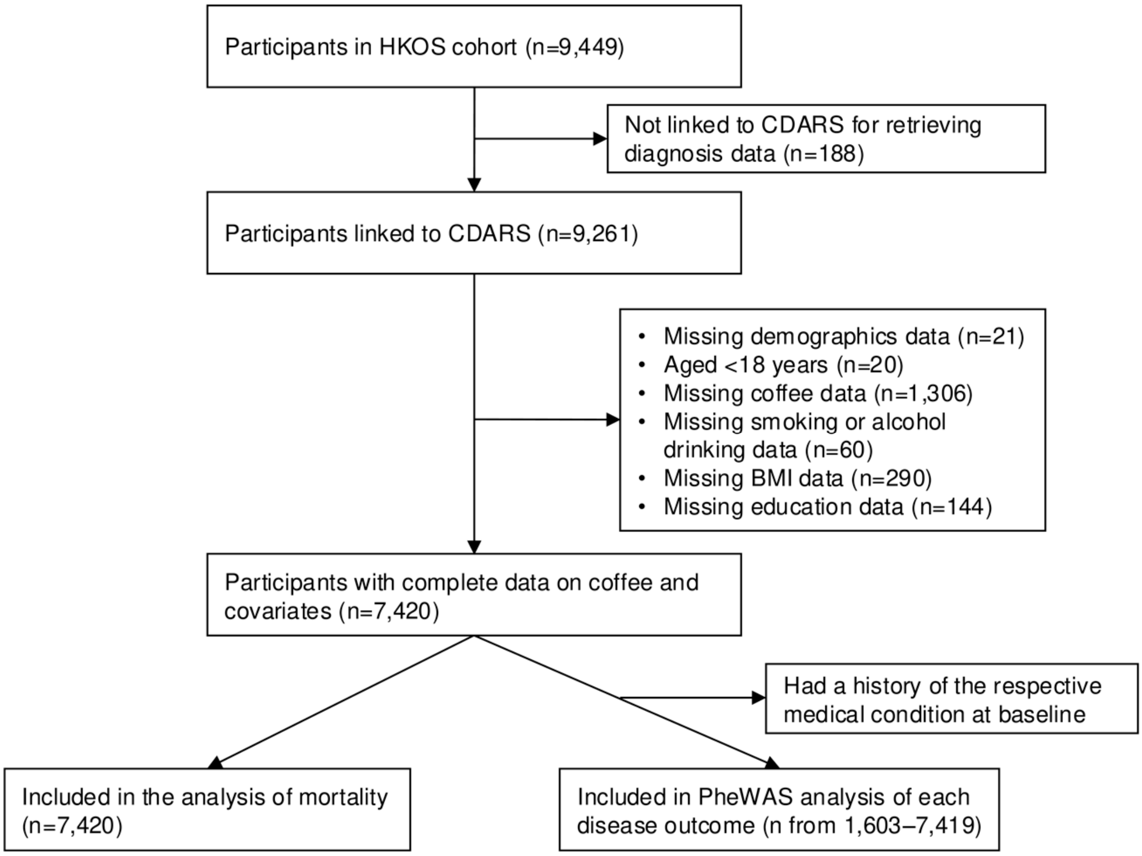

Supplementary Figure S1. Flowchart of sample selection.

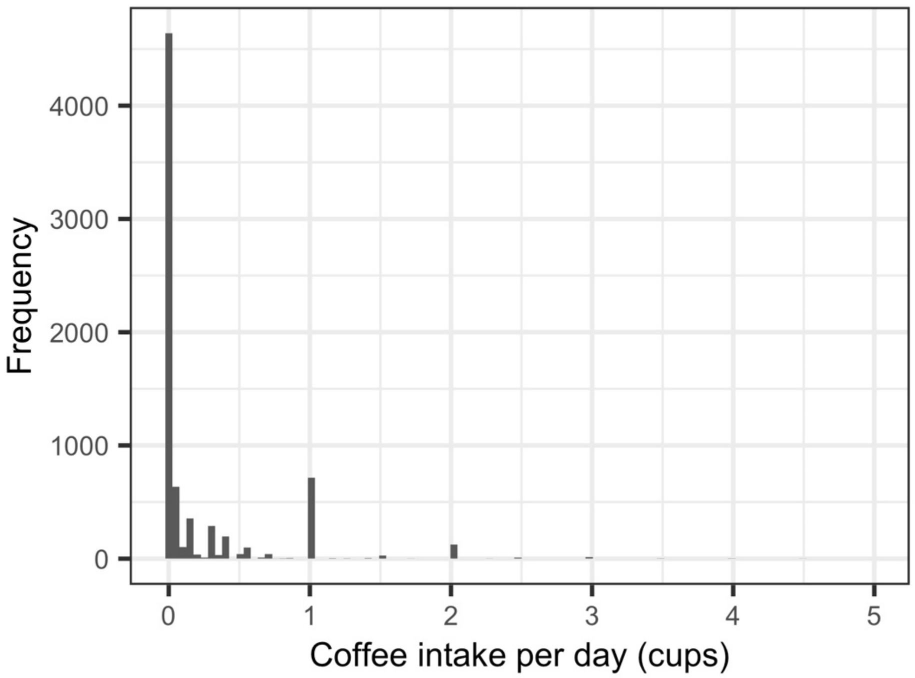

Supplementary Figure S2. Histogram of the number of cups of coffee intake in the Hong Kong Osteoporosis Study.

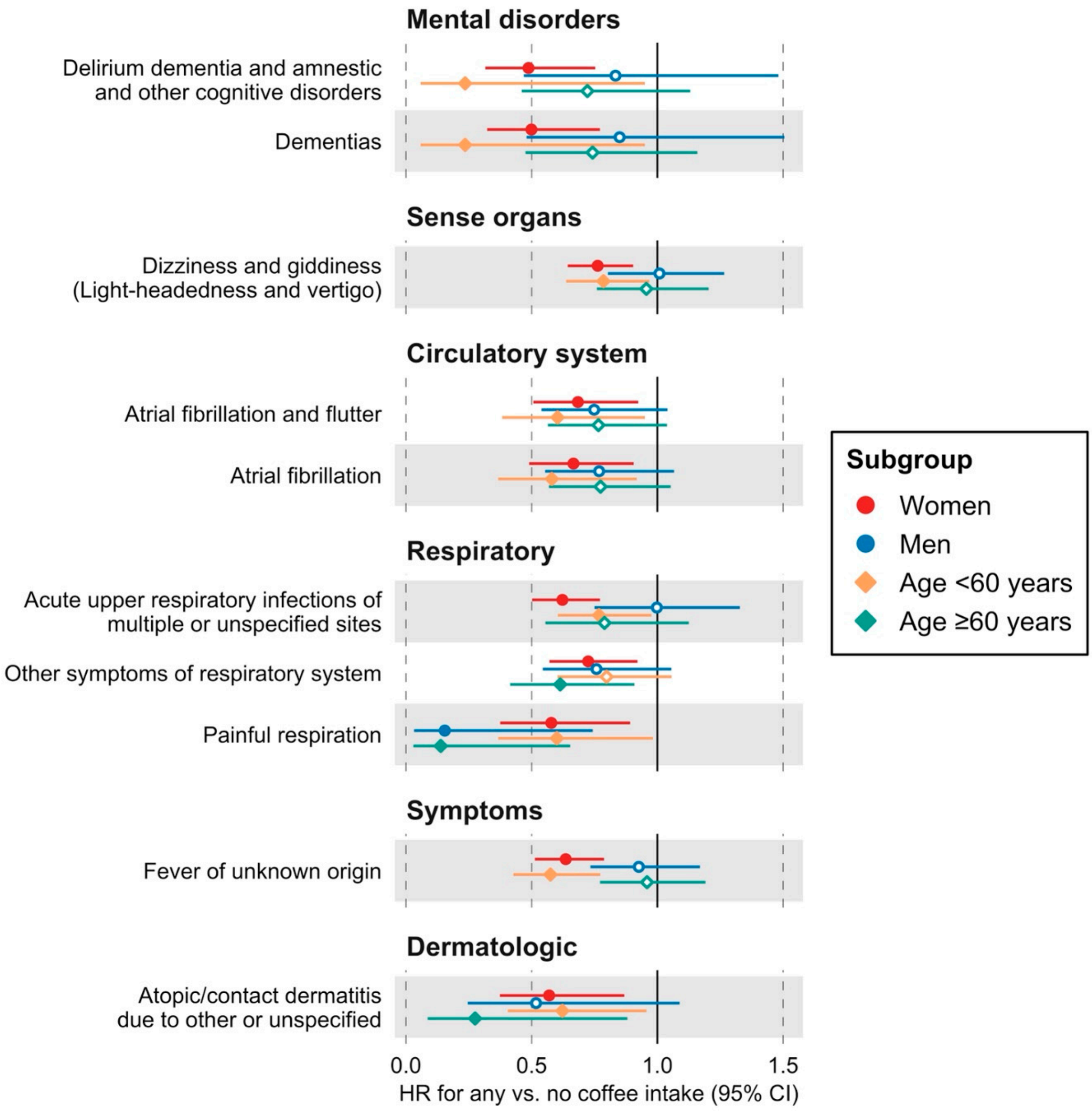

Supplementary Figure S3. Subgroup analysis for the association between any coffee intake and the 10 top identified phecode diagnoses at FDR <0.05.

All models were adjusted for age, sex, body mass index, smoking, alcohol drinking, and education. Filled symbols indicate $p<0.05$. CI, confidence interval; HR, hazard ratio.

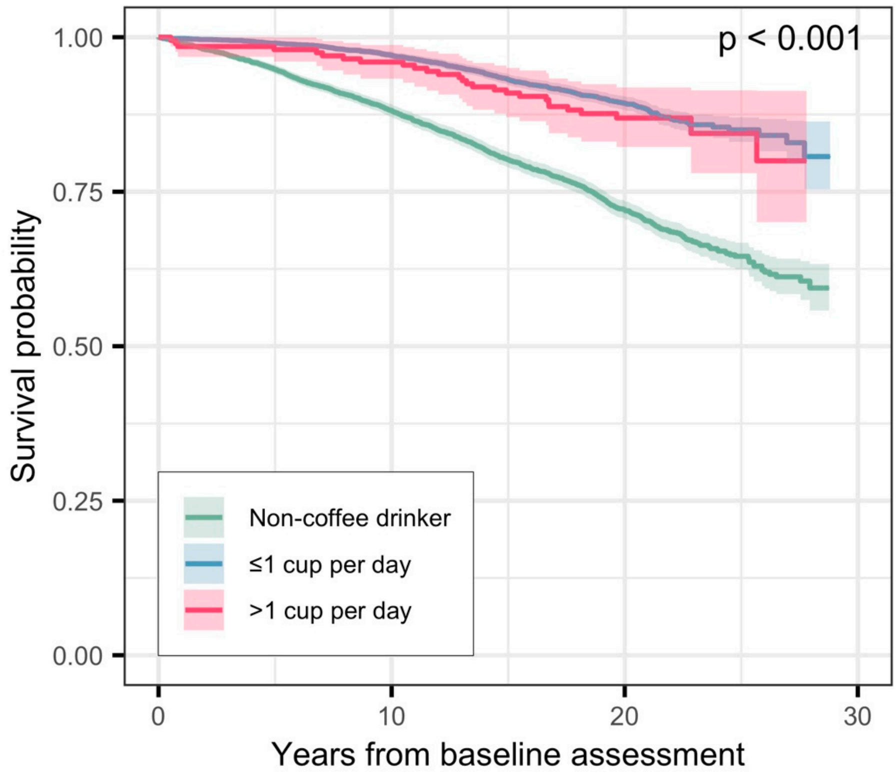

Supplementary Figure S4. Survival probability stratified by daily coffee consumption. P-value was calculated based on log-rank test.

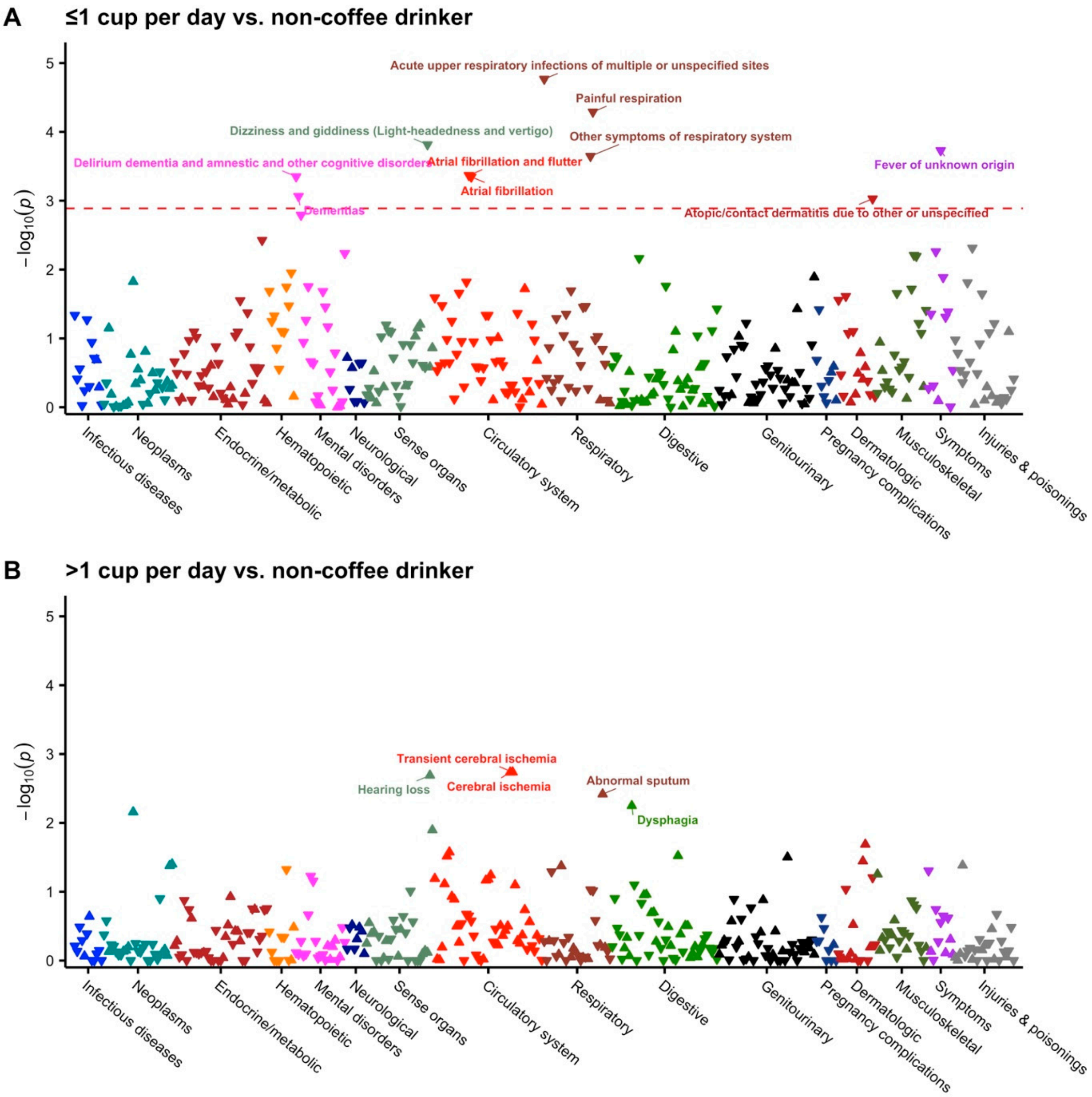

Supplementary Figure S5. Manhattan plot for the phenome-wide analysis of coffee intake categories on disease outcomes.

(A) Estimates for ≤1 cup of coffee per day vs. no coffee intake. Labeled symbols are the diseases significantly associated with coffee intake at FDR <0.05. The dotted red line indicates the significance threshold at FDR=0.05. **(B)** Estimates for >1 cup of coffee per day vs. no coffee intake, of which none reached the threshold of FDR <0.05. Labeled symbols are the top five disease outcomes with the smallest p-values. In both panels, all estimates were calculated based on Cox models adjusted for age, sex, body mass index, smoking, alcohol drinking, and education. Symbols pointing upwards indicate positive associations (hazard ratio >1), whereas symbols pointing downwards indicate inverse associations (hazard ratio <1).

Supplementary Table S1. ICD-9 codes used to define the diseases selected from the literature.

Disease outcomes	ICD-9 codes
Cardiovascular diseases	
Any cardiovascular disease	390-459
Coronary heart disease	410-414, 429.2, 429.71, 429.79
Stroke	430-438
Cancer	
Any cancer	140-208
Prostate cancer	185
Liver cancer	155
Oral cancer	140-149
Non-melanoma skin cancer	173
Liver and gastrointestinal outcomes	
Non-alcoholic fatty liver disease	571.8
Cirrhosis	571.2, 571.5, 571.6, 456.0, 456.1, 456.20, 456.21, 567.0, 567.2, 567.21, 567.29, 567.8, 567.9, 572.2, 572.4, 789.5
Gallstone disease	574
Metabolic conditions	
Type 2 diabetes	250
Renal stones	592.0
Gout	274.0, 274.1, 274.81, 274.89, 274.9
Neurological outcomes	
Parkinson's disease	332, 333.0
Depression	296.20-296.26, 296.30-296.36, 300.4, 311
Alzheimer's disease	331.0
Musculoskeletal outcomes	
Osteoporotic fracture	805, 812-814, 820
Hip fracture	820

Supplementary Table S2. Sample characteristics by coffee intake categories.

Characteristic	Non-coffee drinker	≤1 cup per day	>1 cup per day	*p*
No. of individuals	4,006	3,215	199	
Age, mean (SD)	57.95 (16.26)	47.56 (15.58)	47.66 (14.96)	<0.001
Sex, n (%)				<0.001
Women	2978 (74.3)	2283 (71.0)	94 (47.2)	
Men	1028 (25.7)	932 (29.0)	105 (52.8)	
Body mass index, mean (SD)	22.79 (3.61)	22.78 (3.63)	23.00 (3.61)	0.707
Ever-smoker (%)	473 (11.8)	380 (11.8)	63 (31.7)	<0.001
Ever-alcohol drinker (%)	377 (9.4)	419 (13.0)	53 (26.6)	<0.001
Education level, n (%)				<0.001
Primary or below	1772 (44.2)	624 (19.4)	18 (9.0)	
Secondary	1471 (36.7)	1409 (43.8)	103 (51.8)	
College or university	763 (19.0)	1182 (36.8)	78 (39.2)	
Coffee intake, cup/day, mean (SD)	0.00 (0.00)	0.35 (0.38)	2.13 (0.89)	<0.001
Died during follow-up, n (%)	1166 (29.1)	365 (11.4)	27 (13.6)	<0.001

[a] *P*-values were based on one-way analysis of variance for continuous variables and χ^2-tests for categorical variables.

Supplementary Table S3. Subgroup analysis for the association between any coffee intake and the top identified phecode diagnoses.

Phecode	Description	Subgroup	N[a]	Incident cases	HR (95% CI)[b]	p	$p_{interaction}$[c]
Mental disorders							
290	Delirium dementia and amnestic and other cognitive disorders	Women	5,040	187	0.49 (0.32, 0.75)	1.2×10^{-3}	0.191
		Men	1,896	68	0.83 (0.47, 1.48)	0.535	
		Age <60 years	4,411	12	0.24 (0.06, 0.95)	0.042	0.463
		Age ≥60 years	2,525	243	0.72 (0.46, 1.13)	0.154	
290.1	Dementias	Women	5,039	184	0.50 (0.32, 0.77)	0.002	0.172
		Men	1,894	66	0.85 (0.48, 1.51)	0.577	
		Age <60 years	4,411	12	0.24 (0.06, 0.95)	0.042	0.441
		Age ≥60 years	2,522	238	0.74 (0.48, 1.16)	0.190	
Sense organs							
386.9	Dizziness and giddiness (Light-headedness and vertigo)	Women	5,231	701	0.76 (0.64, 0.90)	1.8×10^{-3}	0.895
		Men	1,981	242	1.01 (0.80, 1.27)	0.944	
		Age <60 years	4,424	380	0.79 (0.64, 0.97)	0.024	0.876
		Age ≥60 years	2,788	563	0.96 (0.76, 1.20)	0.703	

Circulatory system							
427.2	Atrial fibrillation and flutter	Women	4,957	281	0.68 (0.51, 0.92)	0.013	0.553
		Men	1,859	200	0.75 (0.54, 1.04)	0.085	
		Age <60 years	4,288	87	0.60 (0.38, 0.95)	0.029	0.355
		Age ≥60 years	2,528	394	0.77 (0.56, 1.04)	0.086	
427.21	Atrial fibrillation	Women	4,950	273	0.67 (0.49, 0.91)	0.010	0.416
		Men	1,853	194	0.77 (0.55, 1.07)	0.115	
		Age <60 years	4,288	86	0.58 (0.37, 0.92)	0.020	0.303
		Age ≥60 years	2,515	381	0.77 (0.57, 1.05)	0.103	

Supplementary Table S3. (*continued*).

Phecode	Description	Subgroup	N[a]	Incident cases	HR (95% CI)[b]	p	$p_{interaction}$[c]
Respiratory							
465	Acute upper respiratory infections of multiple or unspecified sites	Women	5,273	429	0.62 (0.50, 0.77)	1.5×10^{-5}	0.350
		Men	2,011	159	1.00 (0.75, 1.33)	0.988	
		Age <60 years	4,427	284	0.77 (0.60, 0.98)	0.031	0.042
		Age ≥60 years	2,857	304	0.79 (0.55, 1.13)	0.191	
512	Other symptoms of respiratory system	Women	5,346	361	0.72 (0.57, 0.92)	0.008	0.408
		Men	2,042	172	0.76 (0.54, 1.06)	0.101	
		Age <60 years	4,492	210	0.80 (0.60, 1.06)	0.115	0.095
		Age ≥60 years	2,896	323	0.61 (0.41, 0.91)	0.015	
512.2	Painful respiration	Women	5,104	114	0.58 (0.37, 0.89)	0.013	0.013
		Men	1,923	38	0.15 (0.03, 0.74)	0.020	
		Age <60 years	4,357	70	0.60 (0.37, 0.98)	0.042	0.026
		Age ≥60 years	2,670	82	0.14 (0.03, 0.65)	0.013	
Symptoms							
783	Fever of unknown origin	Women	5,347	498	0.64 (0.51, 0.79)	3.6×10^{-5}	0.090

		Men	2,051	278	0.93 (0.73, 1.17)	0.519	
		Age <60 years	4,492	195	0.57 (0.43, 0.77)	2.5×10^{-4}	0.108
		Age ≥60 years	2,906	581	0.96 (0.77, 1.19)	0.704	
Dermatologic							
939	Atopic/contact dermatitis due to other or unspecified	Women	5,231	113	0.57 (0.37, 0.87)	0.009	0.549
		Men	2,030	54	0.52 (0.25, 1.09)	0.082	
		Age <60 years	4,398	90	0.62 (0.40, 0.96)	0.031	0.343
		Age ≥60 years	2,863	77	0.28 (0.09, 0.88)	0.030	

Note: The listed 10 phecodes are those associated with coffee intake (any vs. no) in the phenome-wide analysis at FDR <0.05.

[a] Number of individuals without the medical condition at baseline.

[b] All models were adjusted for age, sex, body mass index, smoking, alcohol drinking, and education.

[c] *P*-value for the multiplicative interaction term between coffee intake and sex/age.

Supplementary Table S4. Association between any coffee intake and specific diseases selected from the literature.

Disease	N[a]	Incident cases	HR (95% CI)[b]	p	FDR
**Alzheimer's disease**	**7,404**	**159**	**0.49 (0.31, 0.77)**	**2.1×10^{-3}**	**0.024**
Prostate cancer	2,051	83	1.75 (1.10, 2.77)	0.017	0.13
Type 2 diabetes	7,206	1,047	0.89 (0.78, 1.01)	0.079	0.45
Gout	7,377	359	0.83 (0.66, 1.06)	0.133	0.49
Any cancer	7,260	977	1.10 (0.96, 1.26)	0.167	0.49
Depression	7,347	368	0.86 (0.68, 1.07)	0.172	0.49
Osteoporotic fracture	7,066	616	0.90 (0.75, 1.08)	0.263	0.65
Non-melanoma skin cancer	7,415	33	1.52 (0.71, 3.24)	0.282	0.65
Parkinson's disease	7,407	89	0.79 (0.49, 1.29)	0.348	0.73
Coronary heart disease	7,201	778	0.93 (0.80, 1.09)	0.388	0.74
Leukaemia	7,420	21	1.39 (0.55, 3.52)	0.488	0.78
Endometrial cancer	7,415	28	1.32 (0.60, 2.88)	0.489	0.78
Hip fracture	7,257	299	0.91 (0.69, 1.20)	0.508	0.78
Oral cancer	7,406	29	0.78 (0.35, 1.74)	0.547	0.79
Cirrhosis	7,399	68	1.14 (0.67, 1.92)	0.629	0.81
Stroke	7,278	823	0.96 (0.83, 1.12)	0.631	0.81
Any cardiovascular disease	6,677	3,206	0.98 (0.91, 1.06)	0.677	0.82
Liver cancer	7,416	55	1.10 (0.63, 1.94)	0.734	0.83
Non-alcoholic fatty liver disease	7,414	97	1.07 (0.70, 1.63)	0.757	0.83
Gallstone disease	7,327	212	0.98 (0.73, 1.33)	0.920	0.96
Renal stones	7,394	56	0.99 (0.57, 1.73)	0.974	0.97

[a] Number of individuals without the medical condition at baseline.

[b] All models were adjusted for age, sex, body mass index, smoking, alcohol drinking, and education.

Supplementary Table S5. Association between any coffee intake and the top identified phecode diagnoses after excluding the first 2 years of follow-up.

Phecode	Description	N[a]	Incident cases	HR (95% CI)[b]	*p*
Mental disorders					
290	Delirium dementia and amnestic and other cognitive disorders	6838	233	0.57 (0.40, 0.80)	1.2×10^{-3}
290.1	Dementias	6835	229	0.58 (0.41, 0.82)	2.0×10^{-3}
Sense organs					
386.9	Dizziness and giddiness (Light-headedness and vertigo)	7075	889	0.74 (0.63, 0.86)	6.2×10^{-5}
Circulatory system					
427.2	Atrial fibrillation and flutter	6705	444	0.67 (0.53, 0.83)	3.5×10^{-4}
427.21	Atrial fibrillation	6694	432	0.67 (0.53, 0.84)	4.4×10^{-4}
Respiratory					
465	Acute upper respiratory infections of multiple or unspecified sites	7141	530	0.68 (0.57, 0.83)	8.0×10^{-5}
512	Other symptoms of respiratory system	7273	509	0.70 (0.58, 0.86)	4.8×10^{-4}
512.2	Painful respiration	6929	146	0.42 (0.28, 0.63)	2.7×10^{-5}
Symptoms					
783	Fever of unknown origin	7290	759	0.74 (0.63, 0.87)	3.7×10^{-4}
Dermatologic					
939	Atopic/contact dermatitis due to other or unspecified	7158	157	0.52 (0.36, 0.74)	3.2×10^{-4}

Note: The listed 10 phecodes are those associated with coffee intake (any vs. no) in the phenome-wide analysis at FDR <0.05.

[a] Number of individuals without the medical condition at baseline.

[b] All models were adjusted for age, sex, body mass index, smoking, alcohol drinking, and education.

Supplementary Table S6. Association between any coffee intake and all-cause mortality after excluding the first 2 years of follow-up.

Subgroup	N	Deaths	HR (95% CI)[a]	*p*
Full sample	7,325	1,463	0.83 (0.73, 0.94)	0.003
Women	5,304	913	0.82 (0.69, 0.97)	0.018
Men	2,021	550	0.83 (0.67, 0.99)	0.048
Age <60 years	4,494	180	0.93 (0.69, 1.27)	0.67
Age ≥60 years	2,831	1,283	0.80 (0.70, 0.92)	0.002

[a] All models were adjusted for age, sex, body mass index, smoking, alcohol drinking, and education.
